# Pre‐Transplant Infection Screening and Vaccination During Prolonged Waiting Periods for Lung Transplantation in Donor‐Limited Asia‐Pacific Settings

**DOI:** 10.1002/resp.70226

**Published:** 2026-02-15

**Authors:** Takashi Hirama, Miki Nagao, Yoshinori Okada

**Affiliations:** ^1^ Division of Organ Transplantation Tohoku University Hospital Sendai Japan; ^2^ Department of Thoracic Surgery Institute of Development, Aging and Cancer, Tohoku University Sendai Japan; ^3^ Department of Clinical Laboratory Medicine Kyoto University Graduate School of Medicine Kyoto Japan

**Keywords:** drug resistance, lung transplantation, microbial sensitivity tests, vaccination, waiting lists

## Abstract

- Donor limitation and prolonged waiting period create a need for time‐based infection screening and vaccination in lung transplant candidates.
- Respiratory cultures at listing are insufficient without repeat cultures and updated susceptibilities during the waiting period.
- Perioperative antimicrobials for multidrug‐resistant gram‐negative rods, moulds, and nontuberculous mycobacteria should be prepared in advance of organ allocation.
- Vaccination should be completed early during the waiting period; live vaccines should be given before transplantation only when indicated and eligible.

Lung transplantation is uniquely vulnerable to infectious complications because the allograft is continuously exposed to the external environment via the airway, while immunosuppression is typically intensive. Early respiratory infections can directly injure the graft, complicate airway healing and contribute to allograft dysfunction, with major implications for survival [1]. Infection risk assessment and peri‐transplant preparation should therefore start before transplantation and continue across the entire waiting period, rather than being confined to the perioperative window.

A recent review has summarised core elements of pre‐transplant infectious disease screening for thoracic transplant candidates (hereafter, ‘candidates’ refers to potential lung transplant recipients) [2, 3, 4]. Such summaries provide an essential baseline. However, donor‐limited settings across parts of the Asia‐Pacific region face an additional practical problem: the waiting period may be prolonged, and infection risk can substantially change over time [5, 6]. Here, “donor‐limited settings” refers to jurisdictions where deceased donation and lung transplant activity remain low, creating a structural imbalance between demand and organ supply and, consequently, prolonged waiting periods. For context, international data for 2024 show that Spain, the United States, Belgium and Austria report 20.8–53.9 actual deceased donors per million population (pmp) and 9.2–13.1 lung transplants pmp, whereas Japan, the Republic of Korea, China and India report 0.8–7.7 donors pmp and 0.2–3.6 lung transplants pmp [7].

Respiratory microbiology and resistance profiles evolve, exposures accumulate through daily life and travel, and vaccination needs shift across seasons and outbreaks. In this context, a time‐based approach becomes critical, with three practical phases: first, establish a clear baseline at listing, second, optimise vaccination early during the waiting period, and third, update respiratory microbiology and perioperative plans up to transplantation so that organ allocation decisions are not delayed by avoidable uncertainty.

Figure 1 summarises a simple three‐step workflow designed for this “long waiting period” reality. Step 1, at listing, establishes baseline infection status and immunity using serology consistent with major guidance [2, 3, 4]. Alongside baseline serologies commonly performed in routine respiratory practice (such as hepatitis B and C and human immunodeficiency virus testing, as well as tuberculosis screening with an interferon‐gamma release assay or a tuberculin skin test) [8], transplant‐specific assessment is crucial for pathogens that may become clinically dominant under potent immunosuppression, including cytomegalovirus, Epstein–Barr virus, and *Toxoplasma gondii*. For example, hepatitis B status should be defined using hepatitis B surface antigen, hepatitis B core antibody, and hepatitis B surface antibody, with nucleic acid testing when indicated to guide vaccination of non‐immune candidates and pre‐transplant nucleos(t)ide analogue therapy for those with active infection or at risk of reactivation [8]. Candidates should also be evaluated to exclude active tuberculosis, and latent tuberculosis infection should be treated before transplantation when feasible using an isoniazid‐ or rifampicin‐based regimen. These results frame post‐transplant prophylaxis and monitoring strategies and help anticipate complications that are uncommon in general respiratory practice.

**FIGURE 1:**
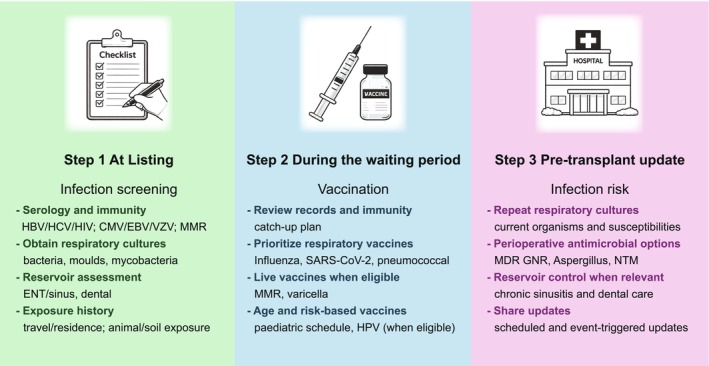
Three‐step workflow for time‐based pre‐transplant infection screening and vaccination in lung transplant candidates in donor‐limited Asia‐Pacific settings. Baseline assessment at listing, vaccination during the waiting period, and pre‐transplant update with repeat respiratory cultures, updated susceptibilities, and perioperative antimicrobial options before organ allocation are summarised. CMV, cytomegalovirus; EBV, Epstein–Barr virus; HBV, hepatitis B virus; HCV, hepatitis C virus; HIV, human immunodeficiency virus; HPV, human papillomavirus; MDR GNR, multidrug‐resistant gram‐negative rods; MMR, measles–mumps–rubella; NTM, nontuberculous mycobacteria; SARS‐CoV‐2, severe acute respiratory syndrome coronavirus 2; VZV, varicella‐zoster virus.

Step 1 also records respiratory microbiology through sputum cultures for bacteria, moulds and mycobacteria [9]. In lung transplantation, respiratory organisms are not a minor detail: they shape perioperative antibacterial and antifungal choices, influence early post‐transplant prophylaxis decisions, and can directly determine the feasibility of an effective perioperative regimen when resistance is present. Assessment at this time should include modifiable reservoirs that can seed post‐transplant infection, particularly sinonasal disease and dental foci [10]. Reservoir control should also include reusable home respiratory devices such as nebulisers and non‐invasive ventilation interfaces, with routine cleaning, disinfection and thorough drying, and targeted culture or replacement when ongoing colonisation suggests device contamination [11]. The purpose is not to create additional barriers to listing, but to identify treatable sources and to plan peri‐transplant management. Finally, listing should capture exposure history (birthplace, travel or residence, and relevant animal or soil exposure) to guide targeted testing for geographically restricted infections [5]. For example, *Strongyloides stercoralis* screening should be considered for candidates with residence or travel in endemic areas, given the risk of hyperinfection after transplantation. This exposure‐driven approach is particularly relevant in the Asia‐Pacific region because endemic infections and travel patterns vary substantially across countries and testing should be tied to clear downstream actions. Core early post‐transplant threats (cytomegalovirus, gram‐negative rods including *Pseudomonas aeruginosa* and *Aspergillus* species) are common across Asia‐Pacific, whereas geographically restricted infections (e.g., tuberculosis and strongyloidiasis) require exposure‐based targeted testing.

Step 2 uses the waiting period to optimise vaccination early, because vaccine responses are often weaker after transplantation. Respiratory vaccines are the priority, including annual influenza vaccination, up‐to‐date SARS‐CoV‐2 vaccination, and pneumococcal vaccination [2, 3, 4]. Where available and appropriate, respiratory syncytial virus (RSV) and recombinant zoster vaccination can be considered in age‐eligible candidates. For age‐eligible candidates who have not completed the series, human papillomavirus (9‐valent) vaccination should be completed before transplantation when feasible. Live vaccines (measles–mumps–rubella and varicella) should be completed before transplantation when indicated and when eligible, ideally with adequate lead time [2]. Eligibility deserves explicit attention because some candidates are already receiving immunosuppressive therapy (e.g., for connective tissue disease), in which case live vaccines may be inappropriate. For children, vaccination should be planned early and updated over time using age‐appropriate and catch‐up schedules [3]. However, several inactivated vaccines can be administered after transplantation if pre‐transplant completion is not feasible.

Step 3 is the phase that most strongly determines real‐world safety in donor‐limited regions: repeat respiratory cultures with updated susceptibilities and securing perioperative antimicrobial options before organ allocation. Multidrug‐resistant gram‐negative rods (including *Pseudomonas aeruginosa* ) [9], moulds (particularly *Aspergillus* species) [12, 13], and nontuberculous mycobacteria (including *Mycobacterium avium* complex and *Mycobacterium abscessus* complex) [14, 15] remain recurring drivers of early infectious risk and therapeutic constraint in lung transplantation. When these organisms are present, perioperative options should not be decided after the graft arrives. They should be prepared in advance using the most recent susceptibility data, with input from infectious diseases teams. This approach also allows centres to address practical constraints (e.g., availability of alternative agents or need for pre‐approval) and to pair antimicrobial plans with reservoir control when relevant (such as optimization of sinonasal disease).

Operationally, scheduled updates and event‐triggered updates help keep risk assessment current throughout the waiting period. Scheduled updates (e.g., every 3–6 months, adapted to local risk and disease trajectory) provide a predictable schedule for repeating cultures, reviewing vaccination status, and reassessing modifiable reservoirs. Event‐triggered updates should occur after exacerbations, hospitalizations, acquisition of a resistant organism, or different culture positivity for moulds or mycobacteria. These updates should be shared between referring clinicians and transplant centres so that changes in risk are reflected promptly in candidacy review, reservoir management, and perioperative antimicrobial planning.

Time‐based workflow does not replace consensus guidance but converts key recommendations into actions suited to a prolonged waiting period. By linking listing assessment to vaccination during the waiting period and to culture‐based updates before organ allocation, transplant teams can reduce avoidable infectious harm before and immediately after lung transplantation, particularly in donor‐limited Asia‐Pacific settings.

## Funding

This work was supported by Japan Society for the Promotion of Science, 23K08287.

## Conflicts of Interest

The authors declare no conflicts of interest.

## Data Availability

The data that support the findings of this study are available from the corresponding author upon reasonable request.
